# A Case Report of Pericardial Effusion with False-Positive Mesothelioma and Adenocarcinoma Markers as the Initial Presentation of Systemic Lupus Erythematous

**DOI:** 10.1155/2022/8081055

**Published:** 2022-11-03

**Authors:** Gita Bhattacharya, Pritha P Gupta

**Affiliations:** ^1^David Geffen School of Medicine at University of California-Los Angeles, Los Angeles, CA, USA; ^2^Department of Cardiology, David Geffen School of Medicine at University of California-Los Angeles, Los Angeles, CA, USA

## Abstract

Pericardial effusion or the accumulation of fluid in the pericardial sac, can result from infectious, malignant, or autoimmune processes such as systemic lupus erythematous (SLE). However, pericardial effusion is infrequently the first presentation of SLE. Here, we describe the case of a 54-year-old African American woman who presented with hypertensive emergency and was found to have pericardial effusion on echocardiogram. Her hypertensive symptoms resolved with medical management and a work up were positive for serum markers of SLE and mesothelioma cell markers (calretinin, CK 5/6) and adenocarcinoma marker MOC31 in the pericardial fluid. Her effusion ultimately improved on high-dose steroid therapy and has not recurred in one year. Given normal pleura and pericardium on computed tomography (CT) imaging and long-term clinical improvement in SLE therapy, we hypothesize that she had false-positive mesothelioma markers in the setting of SLE.

## 1. Introduction

Pericardial effusion results from acute or chronic fluid accumulation within the pericardial sac. Chronic fluid accumulation, commonly associated with malignant or autoimmune processes can result in large asymptomatic pericardial effusions that may only progress to tamponade at volumes as high as one or two liters.

The causes of pericardial diseases are broad and include inflammatory, neoplastic, vascular, congenital, and idiopathic etiologies. In one study of 173 patients undergoing pericardiocentesis, malignancy was by far the most common cause of large, symptomatic pericardial effusions at 33% [1]. Large, asymptomatic pericardial effusions are described less frequently but have been related to malignancy, rheumatoid arthritis, uremia, or unknown causes [2–5]. It is well known that patients with systemic lupus erythematous (SLE) can have pericarditis as a cardiac manifestation of the disease. The incidence of pericarditis in patients already diagnosed with SLE has varied from 25 percent in symptomatic patients identified through routine clinical diagnosis to 50 percent in asymptomatic patients for whom pericarditis was an incidental finding [6]. In a study of 2,390 patients with SLE, African American ethnicity was predictive of new pericarditis [6]. While pericarditis and pericardial effusion have been associated with SLE, pericardial effusion as the initial presentation of SLE is less common.

We are describing a patient who presented with a large pericardial effusion as the first symptom of SLE, whose pericardial fluid was also positive for mesothelioma and adenocarcinoma markers. In the setting of negative CT and MR imaging and her sustained improvement on steroids, we concluded that the positive markers of malignancy were caused by SLE.

## 2. Case Presentation

A 54-year-old African American female with a history of poorly controlled hypertension, daily cigarette smoking (15 pack-years), no prior known asbestos exposure, and alpha-thalassemia anemia was in her usual state of health until she started experiencing progressively worsening blurry vision, fatigue, headache, and orthopnea, which started 3 weeks prior to admission.

**Figure 1:**
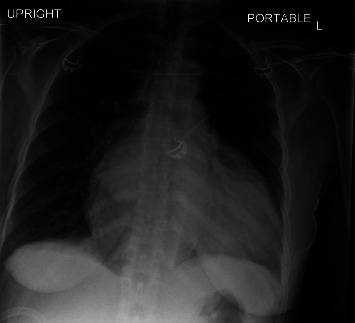
Upright AP CXR obtained on admission shows markedly enlarged cardiac silhouette, mild pulmonary vascular congestion, and bibasilar subsegmental atelectasis.

**Figure 2:**
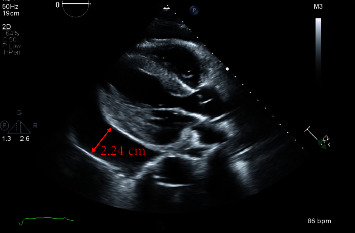
Initial transthoracic echocardiogram obtained on admission shows a large circumferential pericardial effusion measuring 2.24 cm (red line). Cardiac tamponade is present, evinced by early right ventricular diastolic collapse, significant respirophasic variation of mitral inflow and IVC dilation with decreased respiratory variation consistent with elevated right atrial pressure.

**Figure 3:**
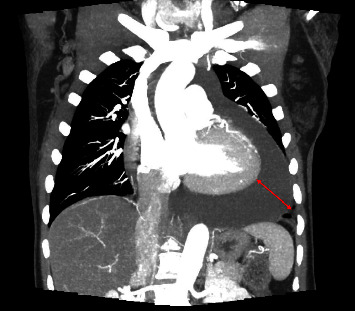
Coronal view of chest CT with angiography obtained due to concern for aortic dissection shows large pericardial effusion (red line) without aortic dissection, pericardial or pleural thickening, or pericardial or pleural masses. Bilateral axillary lymph nodes with normal reniform morphology were present and were thought to be reactive in the setting of large pericardial effusion.

**Figure 4:**
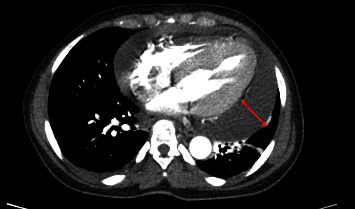
Chest CT with angiography: axial view at midthoracic level shows large pericardial effusion (red line) without pericardial or pleural thickening, or pericardial or pleural masses.

**Figure 5:**
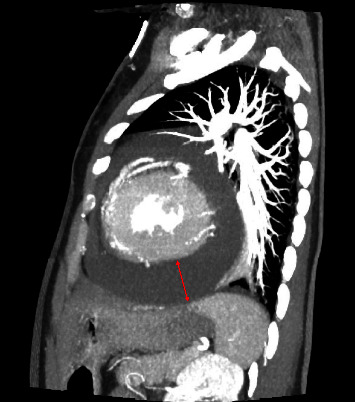
Chest CT with angiography: sagittal view shows large pericardial effusion (red line) without pericardial or pleural thickening or pericardial or pleural masses.

**Figure 6:**
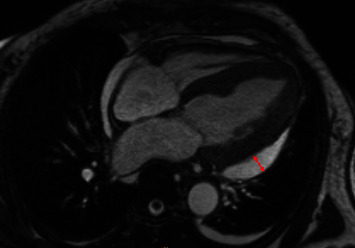
Cardiac MRI 10 days after initial presentation. Axial image captured in mid-diastole shows moderate pericardial effusion (red line) without pericardial or pleural masses.

She was referred to a retina specialist due to concerns for hypertensive retinopathy, where her blood pressure was noted to be 220/130 mmHg and she had cotton wool spots on retinal exam. Due to the physician's concern for hypertensive emergencies, the patient presented to the ED with blood pressures up to 230 mmHg systolic. On examination, the patient did not have sinus tachycardia, pulsus paradoxus, or additional heart sounds. Her jugular venous pressure was elevated to 14 cm above the sternal notch. Bilaterally, the lungs were clear to auscultation. She had no lower extremity edema. Her complete blood count included white blood cell count of 5.70 × 10^9^/L with differential of 62 percent polymorphonuclear cells, 21.2 percent lymphocytes and absolute lymphocyte count of 1.21 × 10^9^/L, 13.9 percent monocytes, and 1.1 percent eosinophils; borderline low red blood cell count of 3.27 × 10^12^/L; low hemoglobin of 7.9 g/dL from a baseline 10 g/dL with MCV 80 *μ*m3; and platelet count of 234 × 10^9^/L. Other significant labs included creatinine of 1.3 mg/dL from a baseline of 1.0 mg/dL, C-reactive protein of 11 mg/L (reference range < mg/L), and erythrocyte sedimentation rate of 110 mm/hr (reference range < 30 mm/hr). Chest X-ray showed a markedly enlarged cardiac silhouette with mild pulmonary vascular congestion (Figure 1). The patient was subsequently admitted to the cardiac care unit for further management. A transthoracic echocardiogram (TTE) showed a large circumferential pericardial effusion measuring 2.24 cm. There was some echocardiographic evidence for tamponade including early right ventricular diastolic collapse, respirophasic variation of mitral inflow and dilatation of the IVC with decreased respiratory variation consistent with elevated right atrial pressure (Figure 2). Her chest computed tomography (CT) with angiogram was negative for aortic dissection. Additionally, there was no pericardial or pleural thickening, or pericardial or pleural masses seen on CT (Figures 3–5) or cardiac MRI (Figure 6). Bilateral axillary lymph nodes with normal reniform morphology were present on CT and MRI and were thought to be reactive in the setting of large pericardial effusion. She was given hydralazine 10 mg intravenously (IV) and oral nifedipine with improvement to 160 s·mmHg systolic in eight hours, then, eventual blood pressure improvement to 120 s/70 s in three days.

Diagnostic and therapeutic pericardiocentesis yielded 1160 mL of straw-colored, nonclotting pericardial fluid, which was sent for infectious and cytologic studies, given her smoking history. A rheumatological work up was also initiated at this time. The patient was started on aspirin 325 mg daily and colchicine 0.6 mg twice daily. Limited echocardiogram on hospital days one and two revealed a stable, small pericardial effusion without echocardiographic evidence of cardiac tamponade.

The patient was found to have markedly elevated serum antinuclear antibody (ANA) of 640 IU/mL, positive anti-Smith antibodies, and hypocomplementemia (low C3 and C4). Rheumatology was consulted for a suspected autoimmune etiology of pericardial effusion. Based on the 2019 ACR/EULAR criteria [7], the patient was classified as having SLE with a score of 15 (greater than 10 is considered diagnostic of SLE) based on positive ANA, low C3 and C4, positive anti-Smith Ab, and presence of pericardial effusion. The decision was made to treat her with high-dose steroids. The patient was started on solumedrol 48 mg IV, followed by two days of 1 g IV daily, and then, 48 mg IV on the day of discharge (hospital day 14).

**Figure 7:**
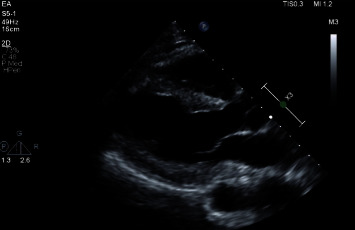
Transthoracic Echocardiogram 4 weeks after initial presentation, showing trace circumferential pericardial effusion. The maximum diastolic dimension of the pericardial effusion seen is 7 mm posteriorly. There are no echocardiographic findings for cardiac tamponade. TEE at a 2-monthfollow-up showed no significant changes.

Two days after starting high-dose IV steroids, a limited echocardiogram revealed a reduction in the size of the pericardial effusion. The patient remained hemodynamically stable throughout the entire admission. Her systolic blood pressures ranged between 130–160 mmHg, and diastolic pressures ranged between 90–100 mmHg in the setting of high-dose IV steroid use. The patient was discharged on a prednisone taper starting at 60 mg daily with a reduction of 10 mg every two weeks. Aspirin was discontinued. Colchicine was continued at 0.6 mg daily for a three-month course. Follow-up TTE four weeks after initial presentation showed stabilization of a small pericardial effusion (Figure 7). She was tapered off prednisone and started on hydroxychloroquine and TTE at 6 months did not show pericardial effusion.

Evaluation of pericardial fluid cytology indicated exudative effusion. Cytology showed few reactive/hyperplastic mesothelial cells and inflammatory cells that were predominantly lymphocytes. Bacterial, viral, and fungal studies were negative. Immunohistochemistry returned positive for calretinin and weakly positive for CK5/6 and MOC31, suspicious for malignant mesothelioma. Evaluation in conjunction with Oncology concluded that the positive calretinin, CK 5/6, and MOC31 were most likely false-positive results, based on her improvement over one month with anti-inflammatory medical treatment and normal pleura and pericardium on CT chest imaging. Thus, the suspicion for malignancy was extremely low and no other work up was completed. Suspicion remained low at one year following presentation, as she remained asymptomatic with normal TTE.

## 3. Discussion

### 3.1. Presentation and Physical Exam

Patients with pericardial effusion may present with nonspecific symptoms such as dyspnea, tachycardia, chest pain, and fatigue. However, pericardial effusion may be asymptomatic if chronic. It is important to evaluate for signs of tamponade on physical exam, which include distant heart sounds, hypotension, elevated jugular venous pressure (Beck's triad), and pulsus paradoxus.

### 3.2. Diagnosis

In cases in which patients are asymptomatic and physical exam is unrevealing, pericardial effusion should be suspected when there is radiographic cardiomegaly without pulmonary congestion. An echocardiogram shows echolucency between the pericardium and epicardium and is considered the best imaging modality for evaluating pericardial effusion and cardiac tamponade. In patients with nondiagnostic TTE in whom suspicion of pericardial effusion remains high, transesophageal echocardiogram (TEE), chest CT, or chest MRI can be helpful in making the diagnosis.

In a young woman with pericardial effusion or pericarditis, a serum ANA test should be considered to rule out SLE. When the etiology of the effusion is less clear, a sampling of the pericardial fluid is required to evaluate the biochemical, immunologic, cytologic, neoplastic, bacterial, fungal, or viral characteristics of the fluid. Pericardial biopsy can be considered in any patient for whom there is still diagnostic uncertainty or in cases where it is convenient to obtain during surgical drainage.

### 3.3. Management

All patients should be observed with hemodynamic monitoring, serial echocardiographic studies, and avoidance of volume depletion. Effusions that are initially stable but progressively enlarge and lead to hemodynamic instability should be treated with pericardial fluid drainage.

### 3.4. Pericardial Disease, SLE, and Mesothelioma

A limited number of case studies have documented pericardial effusion or cardiac tamponade as the initial presentation of SLE [8–11]. All patients in these cases were females under the age of 35, which is consistent with the initial presentation of SLE. Systemic lupus erythematous is known to first present with fever, fatigue, weight loss, malar rash, photosensitivity, discoid lesions, alopecia, arthralgias, or some combination of these symptoms.

Even rarer is the presentation of malignant mesothelioma as SLE. Since 1984, there have been at least four case reports documenting malignant mesothelioma that initially presented and were diagnosed as SLE due to pleural effusion or pericardial effusion combined with clinical features of SLE or positive antibodies [12–15]. Positive mesothelioma markers (including calretinin and CK 5/6) in the effusion cytology, in addition to pleural or pericardial masses on CT chest imaging prompted obtaining biopsies, which confirmed the diagnosis of malignant mesothelioma in each case. While two of the patients' effusions initially improved on steroid therapy intended to treat the initial diagnosis [12, 14], all four patients failed chemotherapy and ultimately died of malignant mesothelioma.

Unlike the patients in these case studies, our patient's effusion has not recurred while on immunosuppressive therapy for her SLE over the course of one year. Additionally, she did not have pleural or pericardial masses on CT imaging. Despite this, her pericardial fluid was positive for calretinin and weakly positive for CK 5/6 and MOC31. Of note, the sensitivity and the specificity of calretinin for malignant mesothelioma are documented as 91 percent and 96 percent, respectively [16]. The sensitivity of CK 5/6 has been documented as 97% [17], and MOC31 has been documented to have high sensitivity and specificity for malignant adenocarcinoma, though sample sizes were small [18]. Given our patient's presentation, we hypothesize that this is a rare instance of false-positive malignancy markers in the setting of lupus, despite the high sensitivity and specificity of these markers.

## 4. Conclusions

In our patient who presented with hypertensive emergency, a large, likely acute-on-chronic pericardial effusion was found in the setting of positive lupus markers, leading to a diagnosis of SLE. Suspicions were raised for mesothelioma when cytology studies returned positive for calretinin and weakly positive for CK 5/6 and MOC31. However, given her improvement on immunosuppressive therapy, it was determined that her effusion was most likely secondary to SLE rather than mesothelioma. Although her presenting pericardial effusion improved and has not recurred, the patient's lupus has progressed over one year, now requiring stronger immunosuppressants (hydroxychloroquine and mycophenolate) in the setting of advanced lupus nephritis. There was no pericardial effusion on TTE performed at the one-year point.

While a handful of case studies have described patients with SLE presenting with pericardial effusion positive for mesothelial cell markers, all described patients were ultimately diagnosed with mesothelioma [12–15], consistent with the high sensitivity and specificity of mesothelioma markers. Importantly, the patients' effusions in these studies were not controlled long-term with immunosuppressives, whereas our patient's effusion has not recurred with management of her SLE. Our patient's case represents a rare instance in which SLE may cause false positivity of mesothelioma and adenocarcinoma markers. Therefore, clinicians should consider the possibility of autoimmune diseases such as lupus causing false-positive mesothelioma or adenocarcinoma markers in patients presenting with autoimmune pericardial effusions who do not show evidence of mesothelioma or adenocarcinoma on imaging.

## Data Availability

The images and labs used to support the findings of this study are included within the article.

## Additional Points

Learning Objectives. Large pericardial effusion may be the initial presenting sign of systemic lupus erythematous. Mesothelioma or adenocarcinoma cell markers can be a false-positive finding in the setting of autoimmune disease.
